# Abnormal Ultrasonographic Findings among Referred Patients with Pain Abdomen in the Radiology Department of a Tertiary Care Center: A Descriptive Cross-sectional Study

**DOI:** 10.31729/jnma.7008

**Published:** 2021-11-30

**Authors:** Smriti Mulmi, Marina Vaidya Shrestha, Sanjeev Pradhan

**Affiliations:** 1 Department of Radiology, Nobei Medical College and Teaching Hospital, Biratnagar, Morang, Nepal; 2 Department of Community Medicine, Kathmandu Medical College, Duwakot, Bhaktapur, Nepal

**Keywords:** *abdomen*, *pain*, *patients*, *radiology*, *ultrasonography*

## Abstract

**Introduction:**

Correct and prompt diagnosis is essential for the appropriate management of patients. Often, children and their caretakers cannot provide a reliable history to allow clinicians to determine the cause of the pain. This study's objective was to find out the prevalence of abnormal ultrasonographic findings among referred patients with pain abdomen in the radiology department of a tertiary care center of Nepal.

**Methods:**

A descriptive cross-sectional study was conducted among patients in the out-patient and emergency department with complaints of abdominal pain from 2021 April 5 to August 30 in a tertiary care hospital. Ethical clearance was taken from IRC (registration no: 423/2021). The convenience sampling method was used. Written informed consent was taken from each study participant. Collected data were entered and analyzed on Statistical Package for the Social Sciences version 20. Point estimate at 95% Confidence Interval was calculated along with frequency and percentage for binary data.

**Results:**

Among 250 patients with pain abdomen referred to the department of radiology, 169 (67.6%) (61.80-73.40 at 95% Confidence Interval) had abnormal ultrasonographic findings. Mean age of the patients was 39.4±17.9 years. Initial clinical diagnosis was in agreement with the abdominal ultrasound diagnosis in 57 (22.8%) patients. For the remaining 193 patients, the diagnosis obtained from abdominal ultrasound differed from the initial clinical diagnosis.

**Conclusions:**

Most of the cases of pain abdomen showed abnormalities in ultrasound. Clinical evaluation should be used together with ultrasound abdomen in order to arrive at a correct diagnosis.

## INTRODUCTION

Abdominal pain is one of the most common symptoms among the patients to visit hospitals.^1^ It can be caused by a variety of diseases ranging from mild and self-limiting to life-threatening diseases.^2^ Approximately 10% of presentations at the Emergency Department are because of acute abdominal pain.^3^ Among these, abdominal ultrasonography (USG) is a non-invasive procedure, which is readily available at most hospitals even during off-hours (weekends, nights and holidays) and may be performed at the bedside.^4^

Abdominal pain is a frequent presentation in any general clinical setting in Nepal with 18% patients which normally had abdominal pain as a feature.^5^ So, rapid diagnosis in time is always needed in the context of our country where computed tomography (CT) scan is not always available in every setting.

The aim of this study is to find out the prevalence of abnormal ultrasonographic findings among referred patients with pain abdomen in the radiology department of a tertiary care center of Nepal.

## METHODS

A descriptive cross-sectional study was conducted from April 5^th^ to August 30^th^ 2021 among patients who came with complaints of abdominal pain in the Radiology department in Nobel Medical College, Biratnagar. Ethical approval was obtained from the Institutional Review Committee (IRC) of Nobel Medical College (IRC registration no: 423/2021). Those patients who were willing to give consent were included in this study. Convenience sampling technique was used and the sample size was calculated by the formula,

The sample size was calculated using the given formula:

n = Z^2^ × p × q / e^2^

  = (1.96)^2^ × 0.50 × (1-0.50) / (0.07)^2^

  = 196

Where,

n= minimum required sample size
Z= 1.96 at 95% Confidence Interval (CI)
p= 50%, for maximum sample size calculation
q= 1-p
e= margin of error, 7%

The minimum required sample size was 196. But, we included 250 referred patients with pain in the abdomen from OPD and the emergency department to the radiology department of our center.

All USG reports were kept in record separately. Day to day Performa was filled up and checked.

Data was entered in the Statistical Package for the Social Sciences version 20. The collected data was analyzed using the same. Descriptive statistics were used and variables were represented in terms of frequency and percentages. Point estimate at 95% CI was calculated.

## RESULTS

**Figure 1:**
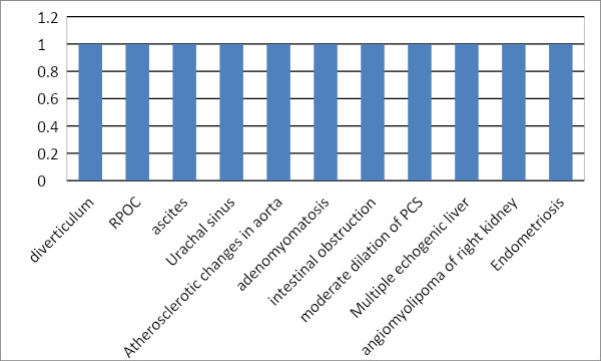
Less common radiological diagnosis of the cases.

Among 250 patients with pain abdomen referred to the department of radiology, 169 (67.6%) (61.8073.40% at 95% Confidence Interval) had abnormal ultrasonographic finding. Based on the ultrasound diagnosis, 20 (8%) were diagnosed as fatty liver, 20 (8%) as ureteric calculi, 15 (6%) as renal cyst, 12 (4.8%) cases each as fibroid and hydronephrosis, 11 (4.4%) each case as nephrolithiasis and cystitis, 8 (3.2%) as bulky uterus, 7 (2.8%) as Acute appendicitis and prostatomegaly, 6 (2.4%) cholelithiasis and mesenteric lymphadenopathy (Table 1 and Figure 1).

**Table 1:** Common radiological diagnosis of the cases seen.

Radiological diagnosis	n (%)
Normal study	81 (32.4)
Fatty liver	20 (8.0)
Ureteric calculi	20 (8.0)
Renal cyst	15 (6.0)
Fibroid	12 (4.8)
Hydronephrosis	12 (4.8)
Cystitis	11 (4.4)
Nephrolithiasis	11 (4.4)
Bulky uterus	8 (3.2)
Prostatomegaly	7 (2.8)
Acute appendicitis	7 (2.8)
Mesenteric lymph nodes	6 (2.4)
Cholelithiasis	6 (2.4)
Gallbladder polyp	4(1.6)
Pancreatitis	4(1.6)
Lump	3(1.2)
Splenomegaly	3(1.2)
PCOD	3(1.2)
Umbilical hernia	2 (0.8)
Heamangioma	2 (0.8)
Renal parencymal disease	2 (0.8)

Most of the patients in this study were females 161 (64.4%) and 89 (35.6%) were males. Age of the patient ranged from 3 years to 87 years. Most of the patients 70 (28%) in our study were in the age group 21-30 years. Mean age of the patients was 39.4±17.9 years. Among these patients, 64 (25.6%) of the patients were from the Brahmin ethnic group followed by the Madeshi group 60 (24%). Most of them believed in Hinduism 234 (93.6%) (Table 2).

**Table 2:** Socio-demographic characteristics of patients (n=250).

Age (years)	n (%)
0-10	7 (2.8)
11-20	20 (8.0)
21-30	70 (28.0)
31-40	46 (18.4)
41-50	44 (17.6)
51-60	21 (8.4)
61-70	28 (11.2)
71-80	10 (4.0)
81-90	4 (1.6)
**Sex**	
male	89 (35.6)
female	161 (64.4)
**Ethnicity**	
Brahmin	64 (25.6)
Madhesi	60 (24.0)
Chettri	57 (22.8)
Janajati	37 (14.8)
Dalit	25 (10.0)
Muslim	7 (2.8)
**Religion**	
Hindu	234 (93.6)
Muslim	5 (2.0)
Buddhist	2 (0.8)
Christian	9 (3.6)

**Figure 2:**
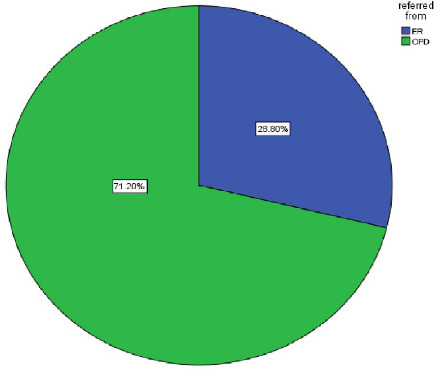
Cases referred to the radiology department (n= 250).

Most of the cases were referred from Opd 178 (71.20%) whereas 72 (28.80%) were from the Emergency department (Figure 2).

The self-reported chief abdominal complaints by the patients were non-specific abdominal pain in 93 (37.2%), epigastric pain 35 (14%), lower abdominal pain 29 (11.6%), and loin pain 25 (10%) (Table 2).

**Table 3:** Chief abdominal complaints of patients.

Complains	n (%)
Non-specific abdominal pain	93 (37.2)
loin pain	35 (14.0)
Lower abdominal pain	29 (11.6)
Epigastric pain	25 (10.0)
Pain in left flank	15 (6.0)
Gynaecological pain	10 (4.0)
UTI compain	10 (4.0)
Pain abdomen	10 (4.0)
Abdominal distension	9 (3.6)
Diffuse pain	8 (3.2)
Right iliac fossa pain	6 (2.4)

Based on clinical examination, 104 (41.6%) cases were diagnosed as acute peptic ulcer disease, 29 (11.6%) as renal calculus, 16 (6.4%) as appendicitis, 12 (4.8%) as acute calculus cholecystitis, 12 (4.8%) as cholelithiasis, 10 (4.0%) as ureteric calculus, 8 (3.2%) as Ovarian cyst, 8 (3.2%) Urinary tract obstruction and others.

Initial clinical diagnosis was in agreement with the abdominal ultrasound diagnosis in 57 (22.8%) patients. For the remaining 193 (77.2%) patients, the diagnosis obtained from abdominal ultrasound differed from the Initial clinical diagnosis. Cases of abdominal ultrasound diagnosis agreed with the Initial clinical diagnosis were renal calculi 18 (7.2 %), Fibroid 11 (4.4%), Acute appendicitis 6 (2.4%), mesenteric lymphadenopathy 5 (2%), Cholelithiasis 4 (1.6%), cystitis 4 (1.6%), Pancreatitis 4 (1.6%), Nephrolithiasis 2 (0.8%), PCOD 1 (0.4%), Urachal sinus 1 (0.4%) and Umbilical hernia 1 (0.4%).

## DISCUSSION

In this study, the mean age of the patients was 39.4 ±17.9 years which was different from the previous study.^7-9^ The cases referred for Ultrasound were mainly from OPD (71.2%) whereas 70% and 97.3% of the cases were from ER in a study done by Lameris W, et al. and by Nural MS, et al.^7,9^ Out of 250 patients in our study 37.2% complained pain abdomen, 14% epigastric pain, 11.6%, lower abdominal pain and 10% loin pain whereas it was described differently in study done by Speets.^8^

In our study, 32.1% cases were normal scans whereas 41% normal scans in Speets.^8^ The initial clinical diagnosis made before Ultrasound diagnosis were appendicitis, cholecystitis, pancreatitis, umbilical hernia and others. A similar initial diagnosis was presented by Nural MS, et al.^9^

This study revealed that 77.2% of initial diagnosis did not match with ultrasound diagnosis. This ratio is only 23% in study by Nural MS, et al.^9^ So the cases should be diagnosed in accordance with the ACR appropriateness of imaging modality. It is considered that Ultrasonography is the initial imaging study of choice for evaluating patients with acute right upper quadrant pain with high ACR appropriate rating.

The present study had a relatively small number of patients examined. The number of patients was limited as the study was restricted to patients subjected to abdominal US as the first diagnostic imaging method.

## CONCLUSIONS

Most of the cases of pain abdomen will show abnormalities in ultrasound. So, it should be used together with ultrasound abdomen in order to arrive at a correct diagnosis. Abdominal ultrasound was suitable for the diagnosis of patients with abdominal symptoms. It is recommended that Ultrasound diagnostic imaging be performed as first line diagnosing modality for patients and further referral to respective departments can treat patients in less time.
